# Agreement of Immunoassay and Tandem Mass Spectrometry in the Analysis of Cortisol and Free T4: Interpretation and Implications for Clinicians

**DOI:** 10.1155/2010/234808

**Published:** 2010-07-12

**Authors:** Rochelle E. Tractenberg, Jacqueline Jonklaas, Steven J. Soldin

**Affiliations:** ^1^Departments of Neurology; Biostatistics, Bioinformatics & Biomathematics and Psychiatry, Georgetown University Medical Center, Washington, DC 20007, USA; ^2^Division of Endocrinology, Georgetown University Medical Center, Washington, DC 20007, USA; ^3^Bioanalytic Core Laboratory, General Clinical Research Center, and Departments of Pharmacology and Medicine, Georgetown University Medical Center, Washington, DC 20007, USA

## Abstract

*Objective*. To quantify differences in results obtained by immunoassays (IAs) and tandem mass spectrometry (MSMS) for cortisol and free thyroxine (FT4). *Design* & *Patients*. Cortisol was measured over 60 minutes following a standard ACTH stimulation test (*n* = 80); FT4 was measured over time in two cohorts of pregnant (*n* = 57), and nonpregnant (*n* = 28) women. *Measurements*. Samples were analyzed with both IA and MSMS. *Results*. Results for cortisol by the two methods tended to agree, but agreement weakened over the 60-minute test and was worse for higher (more extreme) concentrations. The results for FT4 depended on the method. IA measurements tended to agree with MSMS measurements when values fell within “normal levels”, but agreement was not constant across trimester in pregnant women and was poorest for the extreme (low/high) concentrations. Correlations between MSMS measurements and the difference between MSMS and IA results were strong and positive (0.411 < *r* < 0.823; all *P* < .05). *Conclusions*. IA and MSMS provide different measures of cortisol and FT4 at extreme levels, where clinical decision making requires the greatest precision. Agreement between the methods is inconsistent over time, is nonlinear, and varies with the analyte and concentrations. IA-based measurements may lead to erroneous clinical decisions.

## 1. Introduction

In this paper, we compare the agreement [1–7] of immunoassay (IA) and tandem mass spectrometry (MSMS) in the measurement of the concentrations of two analytes, cortisol, and free T4. Both analytes are critical for normal growth and development, and survival. Thyroid hormone regulates the expression of multiple genes and is necessary to maintain normal function in virtually all organ systems of the body [8]. 

 Cortisol regulates normal responses to stress, and is important for vascular reactivity, carbohydrate metabolism, and immune function [9]. Cortisol measurement is the basis for diagnosing adrenal insufficiency or conditions of glucocorticoid excess. Mismeasurement of cortisol could lead to withholding treatment for patients with adrenal insufficiency or Cushings syndrome, which could have life-threatening consequences. Similarly, incorrect diagnosis of these conditions exposes patients to the toxic side effects of steroid or steroid-lowering therapies, without any expected benefit. FT4 is one of the analytes used to titrate the treatment of hypothyroidism and hyperthyroidism. Erroneous analyte values could lead to mismanagment of these disorders. This is a particular concern in vulnerable populations such as the young, the elderly, those who are pregnant, those suffering from cardiac disease, or those who have thyroid cancer. Although FT4 is interpreted in conjunction with a thyroid stimulating hormone (TSH) level, there are circumstances under which TSH measurements are unreliable [10]. It has also been suggested that during pregnancy a decreased FT4 concentration, not an elevated TSH, specifically places a fetus at risk [11–13]. Thus accurate determination of FT4 will best serve the clinician who wishes to make an informed clinical decision.

Direct/analogue immunoassay (IA) methods for the measurement of FT4 are widely used and are controversial [14–17], and there is discrepancy between different IAs [18–20]. Recent reports of problems with IAs for the measurement of steroids in general have identified lack of specificity as the key cause of unreliability [21, 22]. In this paper, we compare measurements of cortisol and FT4 concentrations made by IA and MSMS plotting the mean versus the difference between two measures of a single analyte. It is important to note here that we seek to quantify the agreement between the two measurement methods and/or their disagreement, and not quantify or correct for the degree of disagreement.

## 2. Methods

### 2.1. Overview

This study represents secondary analyses of previously existing data [24, 25]. There were two different cohorts for whom analyte measurements were already available. Briefly, the cohort for whom cortisol measurements were available consisted of 80 subjects [24] who underwent an outpatient cortrosyn stimulation test as part of an evaluation for adrenal insufficiency [27, 28]. The reference interval for cortisol is 3–21 mg/dL (Soldin SJ, unpublished data). FT4 measurements were available for 28 clinically and biochemically euthyroid nonpregnant women and 98 clinically and biochemically euthyroid pregnant women who underwent testing during one or more trimesters of pregnancy [25]. The reference interval for FT4 is 0.9–1.6 ng/dL in nonpregnant women which is the same as the pediatric reference range [29]. We compared assay results from IA and MSMS using the means-difference plots. We would conclude that the methods agree if we observe no relationship between data points plotted on these axes [2–4]. 

### 2.2. Samples

Samples for cortisol and FT4 were collected in plastic red top tubes (containing clot activator, Vacutainer, manufactured by Becton Dickenson, Franklin Lakes, NJ 07417) and allowed to clot for 20 minutes. The samples were then centrifuged at 4,000 rpm for 10 min, serum separated and immediately stored at minus 80°C until measurement of the analytes by the General Clinical Research Center Bioanalytical Core Laboratory. Cortisol concentrations are reported in *μ*g/dL at baseline and then 30 minutes and 60 minutes after cortrosyn injection. This diagnostic test, which involves injection of synthetic adrenocorticotropic stimulating hormone (cortrosyn), is a common test for diagnosing adrenal insufficiency in clinical situations [28]. FT4 measurements were obtained either in the nonpregnant state or during successive trimesters of pregnancy and are reported in ng/dL.

### 2.3. Assays 

#### 2.3.1. Immunoassays

Cortisol was measured on the DPC Immulite 1000 (Diagnostic Products Corporation, Los Angeles, CA) while FT4s were measured on the Dade RxL Dimension (Dade-Behring Diagnostics, Glasgow, DE).

#### 2.3.2. LC/MS/MS

Both the cortisol and FT4s were assayed as previously published in [17, 20–22]. Both assays were performed using the API-5000 tandem mass spectrometer (Applied Biosystems, Foster City, CA), and both use deuterated internal standards. Cortisol measurement was performed on 200 *μ*L serum [22], while FT4 was measured on a serum ultrafiltrate [17, 20]. Assay values (concentrations of cortisol or FT4) were obtained via IA and MSMS at each of the time points relevant for the two analytes.

#### 2.3.3. Statistical Methods

 Data in each study cohort were analyzed separately using a Bland-Altman (BA) or means-difference plot for the IA-MSMS pairs of measurements. The “summary” of data in the BA plot is reflected in reference lines at zero, representing the ideal mean difference between the two measures, and the values one standard deviation (solid lines) and two standard deviations (short dashed lines) away from zero (long dashed line) on the *Y* axis, with zero being the point on the *Y* axis where the two methods agree perfectly and the lines within one standard deviation bounding the acceptable range for variability in agreement. Pearson correlation coefficients were computed to determine if there was a significant association between the difference between measurements (MSMS-IA) and one of the measurements (MSMS), which would reflect significant disagreement [7].

## 3. Results

The data are shown first for cortisol values at baseline and then 30 and 60 minutes after cortrosyn injection and then for free T4 values sampled in the nonpregnant state and then during the successive trimesters of pregnancy. Tables 1a and 1b present descriptive statistics for cortisol (1A) and FT4 (1B) across the relevant time points.

### 3.1. Cortisol

**Figure 1:**
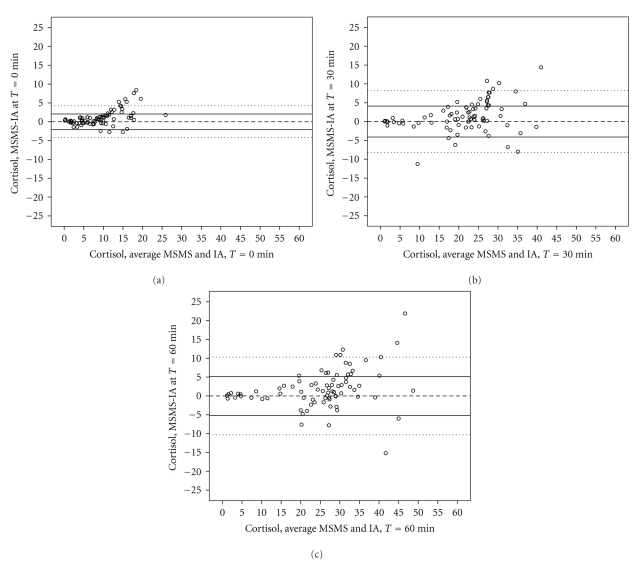
Bland-Altman plot of Cortisol ((mcg/dl) at *T* = 0 (a), *T* = 30 (b), and *T* = 60 (c) minutes) in the cortisol response test: difference MSMS-IA on *Y* axis and average on *X* axis. *N* = 80. Reference line at zero and pairs of lines at ±1SD and ±2SD.

Figure 1 shows the Bland-Altman (BA) plot of the IA and MSMS measurements for the cortisol measurements initially (T0, Figure 1(a)), at 30- (*T*30, Figure 1(b)) and 60-minutes (*T*60, Figure 1(c)).

A clear trend in increasing variance is observed along the *X*-axis (representing the mean of the two measures). Lower concentrations of cortisol measured by IA or MSMS were fairly closely associated, but as the values increase, the divergence becomes larger. This heteroscedasticity within the data at the 30 minute point of the cortisol response test is larger, and reflects a larger range of cortisol values, than were observed at baseline (Figure 1(a)). The same tendency towards increasing variance is apparent in Figure 1(c) (60 minutes after the injection).

Table 2 presents the correlation coefficients estimating the strength of association between the difference between the two measures of cortisol and the MSMS results for cortisol. MSMS results are strongly and positively correlated with the difference.

### 3.2. FT4

**Figure 2:**
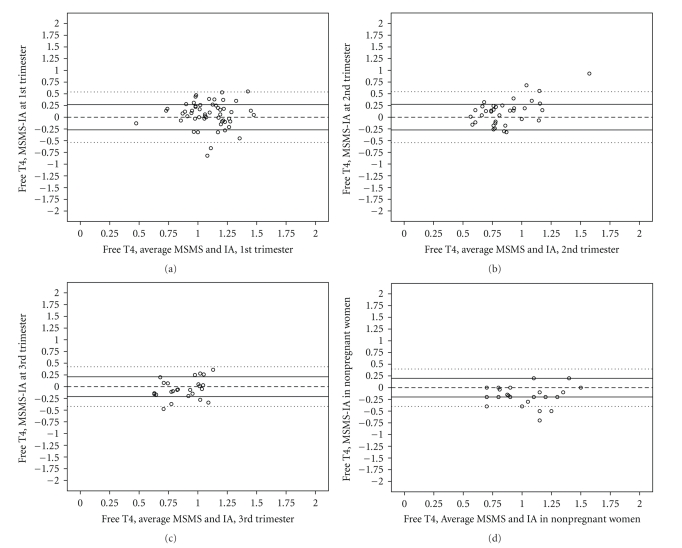
Bland-Altman plots of FT4 (ng/dl), first trimester (T1, 2A), second trimester (T2, 2B), 3rd trimester (T3, 2C), and in nonpregnant (NP, 2D) women. Difference (MSMS-IA) on Y axis and average value from IA and MSMS on X axis. (Pregnant cohort N with both ranges from 59 (T1) to 26 (T3); nonpregnant cohort N is 28.) Reference line at zero and pairs of lines at ±1SD and ±2SD.

Figure 2 shows the Bland-Altman (BA) plots of the IA and MSMS measurements for FT4 measured in the first trimester (T1, 2A), second (T2, 2B) and third trimesters (T3, 2C) for a single group of pregnant women followed over time, and a fourth group of women who were not pregnant (NP, 2D). 

The patterns in the means-difference plots are not as clear cut for FT4 as for cortisol. In the first trimester (T1, Figure 2(a)), the variability in disagreement appears to increase as the average increases (fan shape), while in T2 and T3 the difference itself seems to increase as the average increases (positive slope). For the nonpregnant women, a different pattern is observed, with the majority of points reflecting greater FT4 values from IA relative to those from MSMS. 

Table 3 presents the correlation matrix for the difference in FT4 measured by MSMS and IA with MSMS. 

Strong, positive correlations were observed between the difference between the two methods and MSMS measurements of FT4 over the three trimesters and in the nonpregnant state, suggesting that the patterns reflected in Figures 2(a)–2(d) represent significant differences in the variances of the measurements of FT4 by IA versus MSMS.

## 4. Discussion and Conclusions

This study of two methods to assay FT4 and cortisol over time showed nonlinear disagreement between analytes measured by immunoassay and tandem mass spectrometry. The differences were more dramatic for cortisol than for FT4 but significant correlation coefficients reflected “genuine” trends of increasing variance associating with increased analyte concentration, and over time, for both analytes. In these correlations, we treated MSMS as the standard measurement; IA results are derived through a mathematical formula in the direct/analogue methods used in the great majority (>99%) of clinical laboratories. 

Our results suggest significant variation (heteroscedasticity) and nonequivalence of these two methods. This could simply reflect poor reliability in IA for these analytes but in the case of FT4 it is also perhaps suggestive that the variation in disagreement over trimesters could also have varying causes (i.e., that vary with pregnancy, e.g., heterophilic antibodies, changes in protein binding, etc.). Although the agreement was worse for cortisol, correlations between the difference in results from the two methods and MSMS was significant for both analytes and at each of the time points.

The purpose of this work is not to provide correct/corrected values for the analytes in these populations but rather to highlight areas where interpretation/interpretability might be compromised by unreliability and/or failures of modeling assumptions (such as heteroscedasticity and time-sensitive variability). As noted earlier, this study sought to quantify the agreement between the two measurement methods and/or their disagreement, and not to quantify or correct for the degree of disagreement. The magnitudes of the differences our analyses discovered could be clinically relevant. For example, in the evaluation of adrenal insufficiency they could make the difference between concluding someone has adrenal insufficiency or adrenal sufficiency. In this particular cohort of patients, 11.5% of those tested could be given a different diagnosis (adrenal insufficiency versus adrenal sufficiency) depending on which assay was used to make the diagnosis. Similarly, the differences between the thyroid hormone assays are clinically relevant, particularly in the pregnant population where the thyroid hormone concentration could be most relevant for fetal health [11–13]. Here, our samples did not permit distinction of diagnosis on the basis of IA or MSMS, but this has been found in a current example of FT4 results where 5/46 (10.9%) of patients had IA results for FT4 that could have led to misdiagnosis [20].

Our results suggest that immunoassay and tandem mass spectrometry cannot be considered to yield interchangeable results. The methods did not agree and this disagreement became more extreme and less predictable at higher concentrations of the analytes we studied. As the true concentration becomes more extreme, so does the discrepancy between IA and MSMS results. The implication of this for clinicians is that patients with analyte values at the extremes are more likely to be misdiagnosed/mismeasured when IA is used (see [17, 20, 24, 25]. 

Since the extreme observations are by definition rare outcomes, they might appear to be outliers and not contribute much (particularly in larger samples) to decrease the *R* ^2^. This can artificially inflate confidence in overall fit of a transformation to the data and can lead to undetected incorrect clinical decisions if IA is used and homoscedasticity is assumed. 

Our results demonstrate good agreement between IA and MSMS in the concentration range of least interest—that is, at normal levels [20, 23–26, 29]. More importantly, the methods do not give similar results over time and over the actual concentration of cortisol and, to a lesser but still “genuine” extent, FT4. MSMS has been compared to equilibrium dialysis [25] for analytes including FT4 with excellent agreement.

IA and MSMS methods have been compared elsewhere [17–20], suggesting that IA methods lack specificity and are also subject to interferences from altered binding proteins and nonspecific heterophilic antibodies—which are prevalent during pregnancy (possibly contributing to some of our results). Direct analogue FT4 IA methods have been found to give false results at both the low and high ends of the value continuum [17–20]. Different serum matrices can affect IAs but are far less likely to affect MSMS methods. The latter do not cross-react with similar compounds or metabolites, and so are more specific. Immunoassays, in contrast, may cross-react with similar compounds as well as with many metabolites. The precision or reproducibility of MSMS methods and IA methods are similar, although some IA methods could repeatedly provide a precise, and incorrect value. Drawbacks to MSMS methods are that they require more highly trained operators and generally are slower, with a lower throughput than IAs. Several university, hospital and commercial laboratories are moving from the non-specific immunoassay platforms to the more specific tandem mass spectrometry procedures for steroid and thyroid hormone measurement. This trend is accelerating and the number of laboratories using tandem MSMS and participating in the College of American Pathologists Proficiency Testing Program is increasing significantly each year.

In conclusion, these analyses demonstrate statistically significant disagreement in the measurement of two analytes at levels outside of the reference range by two different assays. It is important for physicians who are making clinical decisions to be aware that the analyte value they are provided with varies depending on the assay used to generate the data. Clinicians may be surprised to discover that the clinical decision they reach may be impacted by the assay employed. The mechanism of action of the MSMS assay relative to that of IA methods support MSMS as the more specific and accurate assay (see also [14, 16–20]). Since disagreement is most pronounced at the extremes (highest/lowest) and clinical intervention is typically based on values in these ranges, we feel that MSMS is the more robust method for assay.

## Figures and Tables

**Table:** (a) Cortisol (mcg/dl) over a 60 minute test period by MS-MS and IA (*N* = 80). Note that agreement is not universal on reference ranges. However, Reference range for random cortisol by IA in most clinical laboratories = 4 –22 mcg/dl ([23]; see also [24]).

	MSMS: MEAN (SD)	IA: MEAN (SD)
cortisol *T* = 0	9.498 (6.08)	8.508 (4.99)
cortisol *T* = 30	21.982 (10.24)	20.553 (8.96)
cortisol *T* = 60	25.758 (12.22)	23.844 (10.74)

**Table:** (b) Free T4 (FT4, ng/dl) over 3 trimesters of pregnancy, and in nonpregnant women. Note that agreement on reference ranges for FT4 in pregnant women is not universal. Kahric-Janicic et al. (2007) [25] suggest ranges between 0.6 and 1.4 ng/dL for FT4 in pregnant women; results vary by trimester for both MSMS and IA. Reference intervals for FT4 in nonpregnant women by tandem mass spectrometry are 0.8–2.1 ng/dL [24]. Ranges for this analyte in nonpregnant women by IA are lower and have a smaller range, typically around 0.7–1.5 ng/dL [26].

	MSMS: MEAN (SD), N	IA: MEAN (SD), N
FT4, trimester 1	1.125 (.23), 59	1.071 (.22), 61
FT4, trimester 2	0.915 (.31), 36	0.795 (.17), 42
FT4, trimester 3	0.863 (.22), 26	0.875 (.18), 35
FT4, nonpregnant women	0.928 (.26), 28	1.102 (.25), 28

**Table 2:** Pearson correlations of difference between methods versus MSMS alone, for Cortisol (*N* = 80). MSMS and (MSMS-IA) correlations reflect significant differences in the variances of the measurements by IA versus MSMS over time.

DIFFERENCE	Cortisol by MSMS, *T* = 0	Cortisol by MSMS, *T* = 30 min	Cortisol by MSMS, *T* = 60 min
*T* = 0 min, MSMS-IA	0.650 *		
*T* = 30 min, MSMS-IA		0.494 *	
*T* = 60 min, MSMS-IA			0.480 *

All Pearson correlation coefficients significant at *P* ≤ .001. * indicates significant differences in the variances of the measurements by IA versus MSMS.

**Table 3:** Pearson correlations of difference between methods versus MSMS alone, for Free T4 (FT4). MSMS and (MSMS-IA) correlations reflect significant differences in the variances of the measurements by IA versus MSMS over time, and for nonpregnant women (NP) measured at one time only. (Pregnant cohort N with both ranges from 59 (T1) to 26 (T3); Nonpregnant cohort N is 28.)

DIFFERENCE	FT4 by MSMS, over time (and for NP)			
	FT4 by MSMS, T1	FT4 by MSMS, T2	FT4 by MSMS, T3	FT4 by MSMS, NP
FT4, T1 MSMS-IA	0.598†*			—
FT4, T2 MSMS-IA		0.823†*		—
FT4, T3 MSMS-IA			0.723†*	—
FT4, NP MSMS-IA	—	—	—	0.411†*

† Pearson correlation coefficient significant at *P* < .05.*indicates significant differences in the variances of the measurements by IA versus MSMS.
